# Supplementary material for: Integrated transcriptomic correlation network analysis identifies COPD molecular determinants
Source: Sci Rep. 2020 Feb 25;10:3361. doi: 10.1038/s41598-020-60228-7 (PMC7042269; doi:10.1038/s41598-020-60228-7)
Supplement: Supplementary file 1 — Supplementary Information. [file 41598_2020_60228_MOESM1_ESM.docx]

# Integrated transcriptomic correlation network analysis identifies COPD molecular determinants

Paola Paci^1*^, Giulia Fiscon^1^, Federica Conte^1^, Valerio Licursi^2^, Jarrett Morrow^3^, Craig Hersh^3^, Michael Cho^3^, Peter Castaldi^3^, Kimberly Glass^3^, Edwin K. Silverman^3^, Lorenzo Farina^4^

## Supplementary Information

This document contains the Supplementary Figures and the descriptions of the Supplementary Tables provided as .xlsx files.

## Supplementary Figures


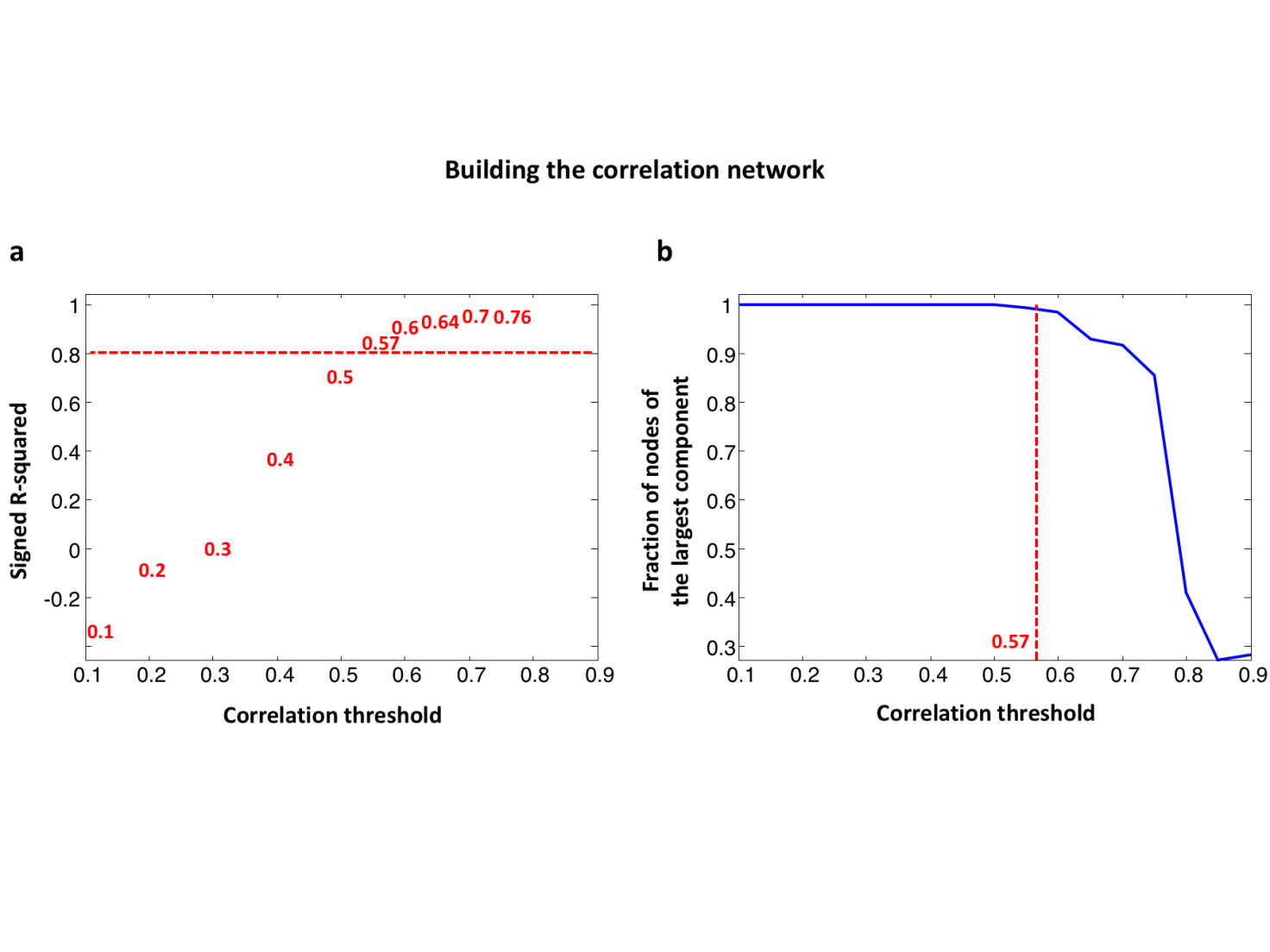


**Supplementary Figure 1. Correlation threshold for COPD network.** (a) Analysis of network topology for various correlation thresholds. The x-axis represents the Pearson correlation threshold varying in the chosen range, while the y-axis represents the scale-free fit index (signed R-squared). The horizontal red line represents the smallest value of signed R-squared (0.8) such that an approximate scale-free topology is reached. (b) Connectivity of the COPD correlation network for various correlation thresholds. The x-axis represents the Pearson correlation threshold varying in the chosen range, while the y-axis represents the fraction of nodes populating the largest component. The dashed red line corresponds to the selected threshold (*ρ* = 0.57 or 98^th^ percentile). Note that y=1 means that all nodes fall in the largest component and thus the network is fully connected; otherwise more components exist.

**
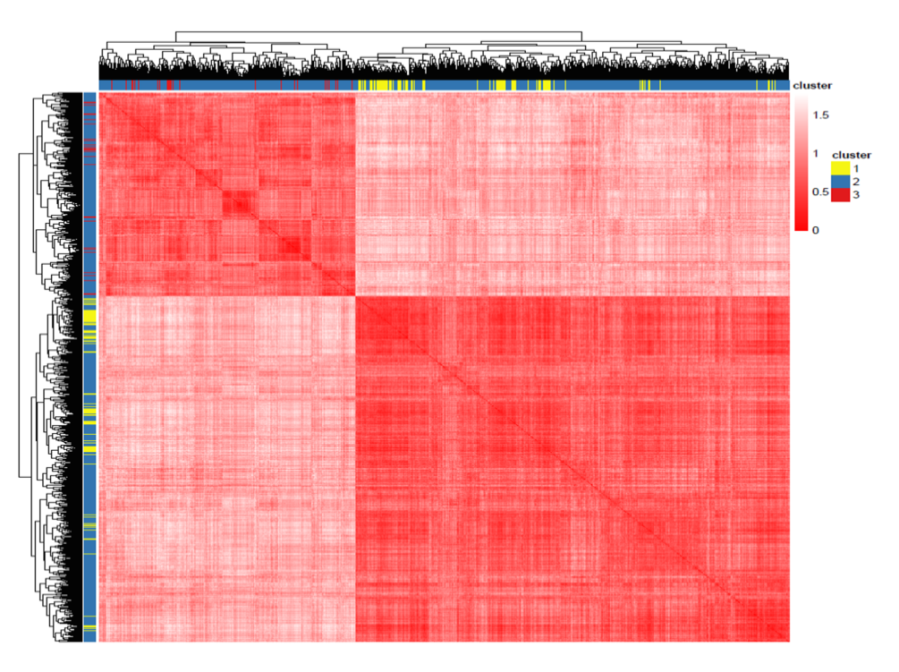
**

**Supplementary Figure 2. Hierarchical clustering for COPD network**. Heat map of the values of the correlation-based dissimilarity where red/white colors indicate high/low values of dissimilarity. Here, the network nodes (rows and columns) have been clustered according to hierarchical clustering with complete agglomeration method and colored according to the k-means cluster assignment.


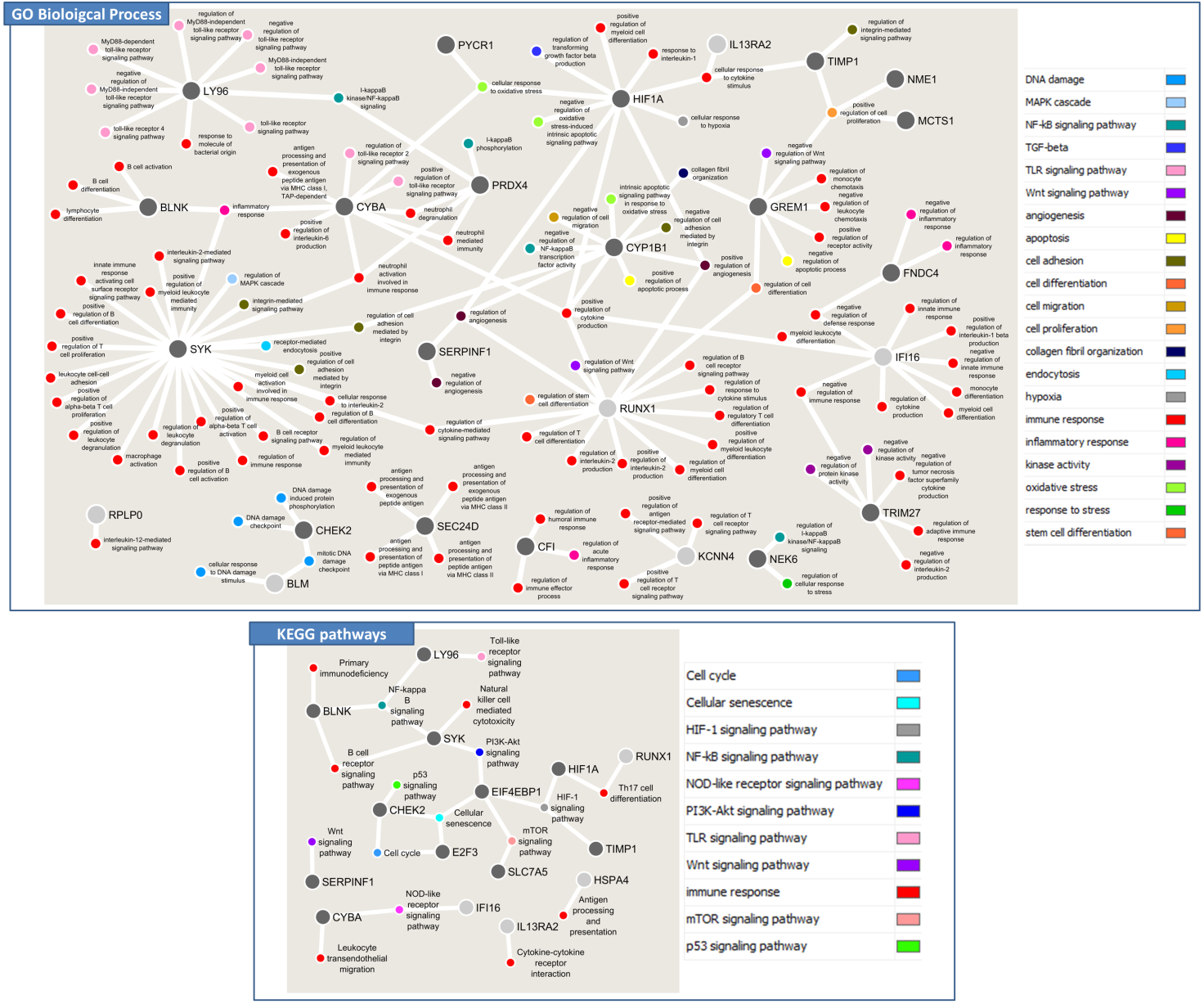


**Supplementary Figure 3. Functional annotation network of switch genes**. In both panel larger dots represent switch genes, smaller dots represent the GO terms (top) and KEGG pathways (bottom) in which they are involved. GO terms and KEGG pathways are coloured according different categories reported in the legend. Switch genes nodes are coloured according to their cluster belonging (i.e., light grey = module 2; dark grey = module 3).


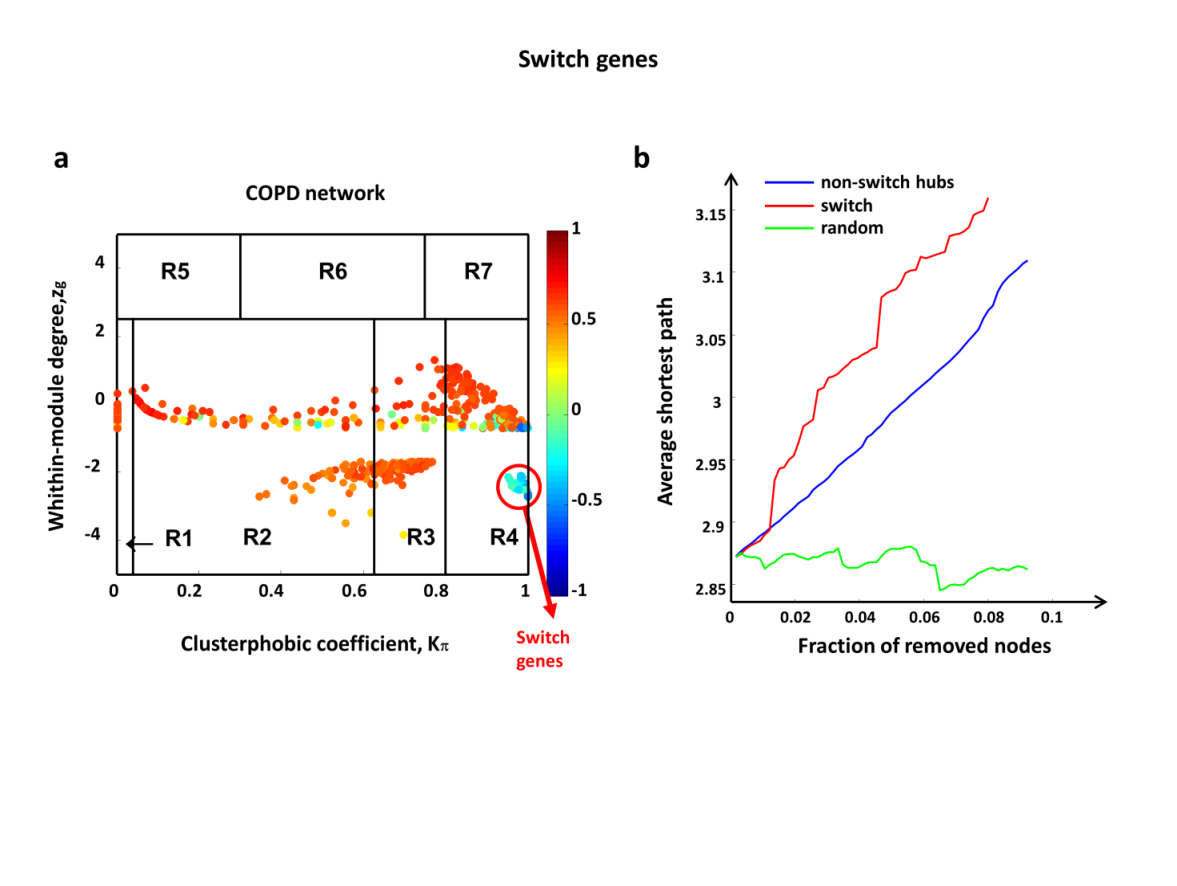


**Supplementary Figure 4.** **COPD switch genes for the test set (GSE76925 dataset).** **(a)** Heat cartography maps of COPD network. Dots correspond to nodes in the networks. Each node is colored according to the value of the APCC between its expression profile and that of its nearest neighbors in the network. **(b)** Robustness for COPD correlation network. Blue curve corresponds to the cumulative deletion of non-switch hubs (i.e. the first 61 hubs that are not switch genes, sorted by decreasing degree); red curve corresponds to the cumulative deletion of the 61 switch genes, sorted by decreasing degree; the green curve corresponds to the cumulative deletion of randomly selected nodes. The x-axis represents the cumulative fraction of removed nodes with respect to the total number of network nodes that is 667 (i.e. x-maximum is 61/667 = 0.09), while the y-axis represents the average shortest path.


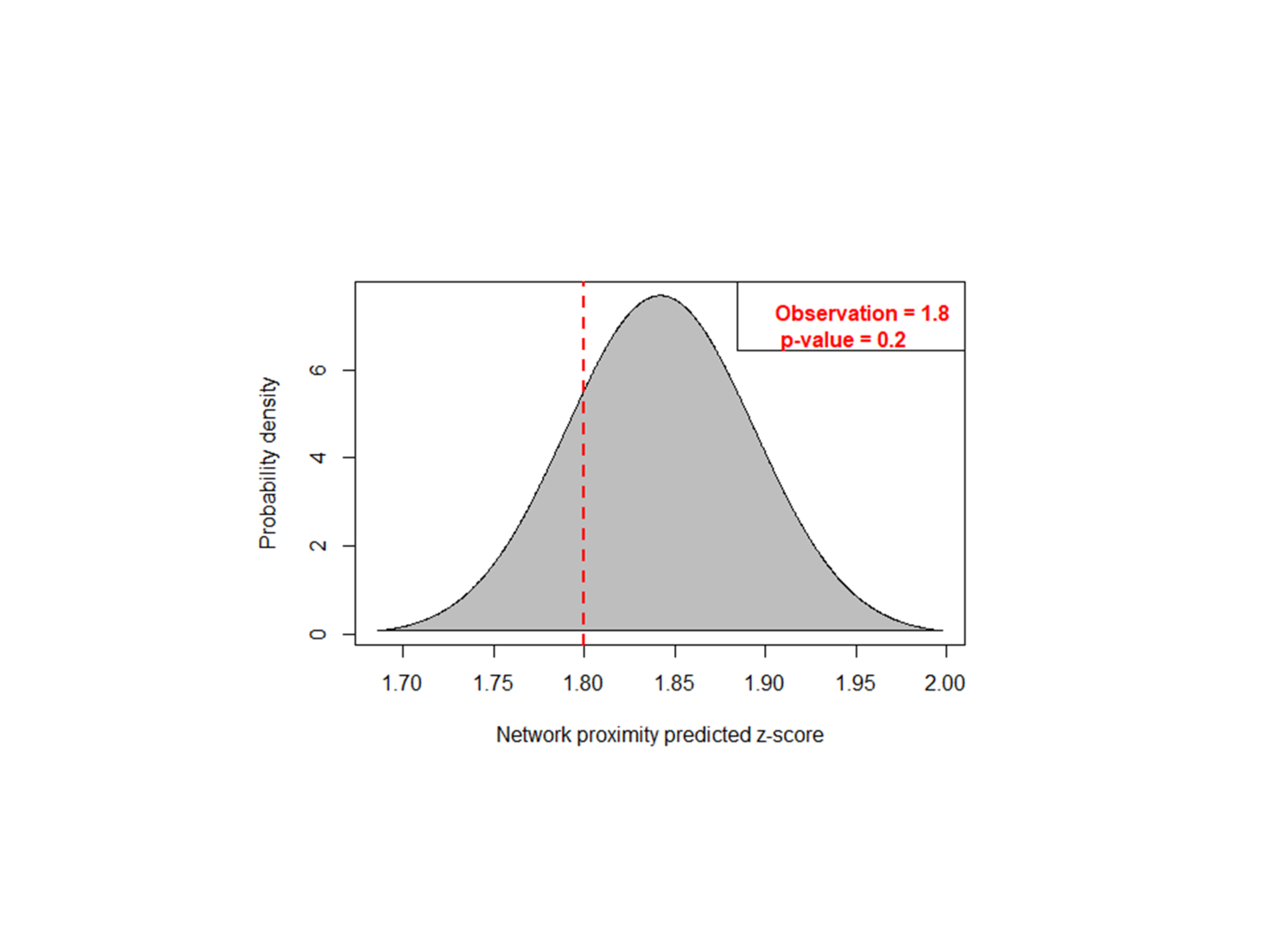


**Supplementary Figure 5. Probability distribution of the network proximity.** The network proximity was computed between switch genes of COPD training set and switch genes of ARDS dataset. The dashed red line corresponds to the observed network proximity measure (p = 1.8) across the lists of switch genes obtained from the two analyzed datasets. The area on the left of the dashed red line represents the probability of observing the test statistic as small as that observed, corresponding to a p-value = 0.2**.**

**
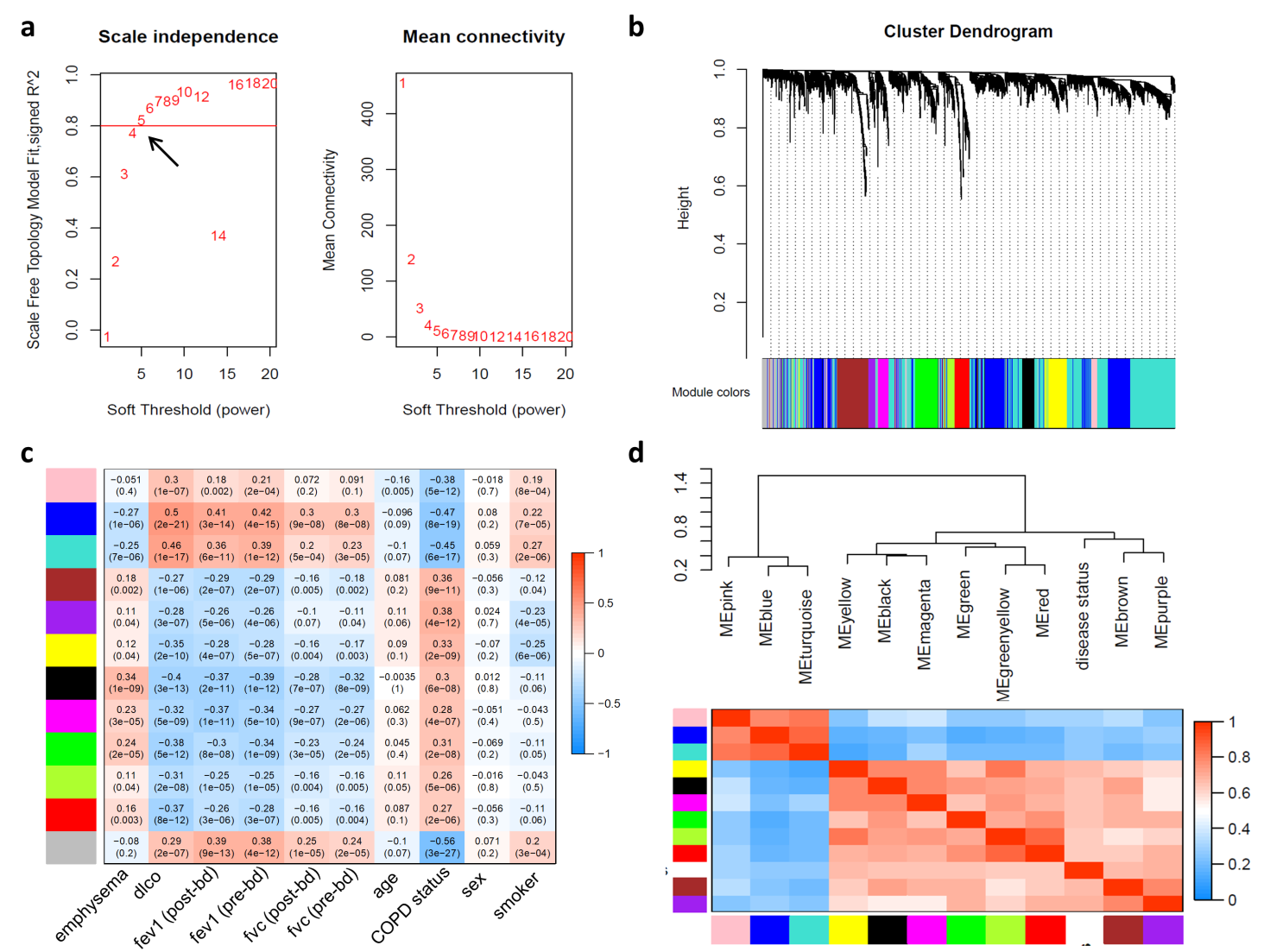
**

**Supplementary Figure 6**. **WGCNA analysis on the COPD training set (GSE47460).** **(a)** Network topology analysis varying the powers of soft-thresholding. Left panel: the scale-free fit index (y-axis) is displayed as a function of the soft-thresholding power (x-axis). Right panel: the mean connectivity (i.e., degree, y-axis) is displayed as a function of the soft-thresholding power (x-axis). **(b)** Hierarchical clustering dendrogram (tree) of genes together with their assigned module colors. Genes are clustered based on the Topological Overlap Matrix (TOM) dissimilarity. Each leaf of the dendrogram corresponds to a gene, branches of the dendrogram that densely group together corresponds to highly co-expressed genes. **(c)** Heatmap of module association with phenotype variables. Each row corresponds to a module eigengene (ME), each column to a phenotype variable. The bottom number in each cell corresponds to the p-value and the top number is the correlation value. The table is color-coded by correlation according to the color legend. COPD status = COPD case-control status, smoker = smoker status (i.e, current, ever, or never), emphysema = the fraction of lung voxels with attenuation less than − 950HU, dlco = the diffusing capacity of carbon monoxide as a percentage of the predicted value, fev1 (post-bd) = the forced expiratory volume in 1sec as a percentage of the predicted value (post bronchodilator test), fev1 (pre-bd) = the forced expiratory volume in 1sec as a percentage of the predicted value (pre bronchodilator test), fvc (post-bd) = the forced vital capacity as a percentage of the predicted value (post bronchodilator test), fvc (pre-bd) = the forced vital capacity as a percentage of the predicted value (pre bronchodilator test). The phenotype variables dlco, fev1 (post-bd and pre-bd), fvc (post-bd and pre-bd), and smoker status decrease with disease status, while emphysema increases with disease. **(d)** Module eigengene dendrogram together with the adjacency heatmap quantifying the module similarity by eigengenes correlation to identify groups of correlated module eigengenes. Top: the hierarchical clustering dendrogram of the eigengenes is displayed where the dissimilarity of module eigengenes MEi, MEj is given by 1 - cor(MEi, MEj). Bottom: the heatmap shows the module eigengene adjacency A_ij_ = 0.5 *(1 + cor(MEi, MEj).


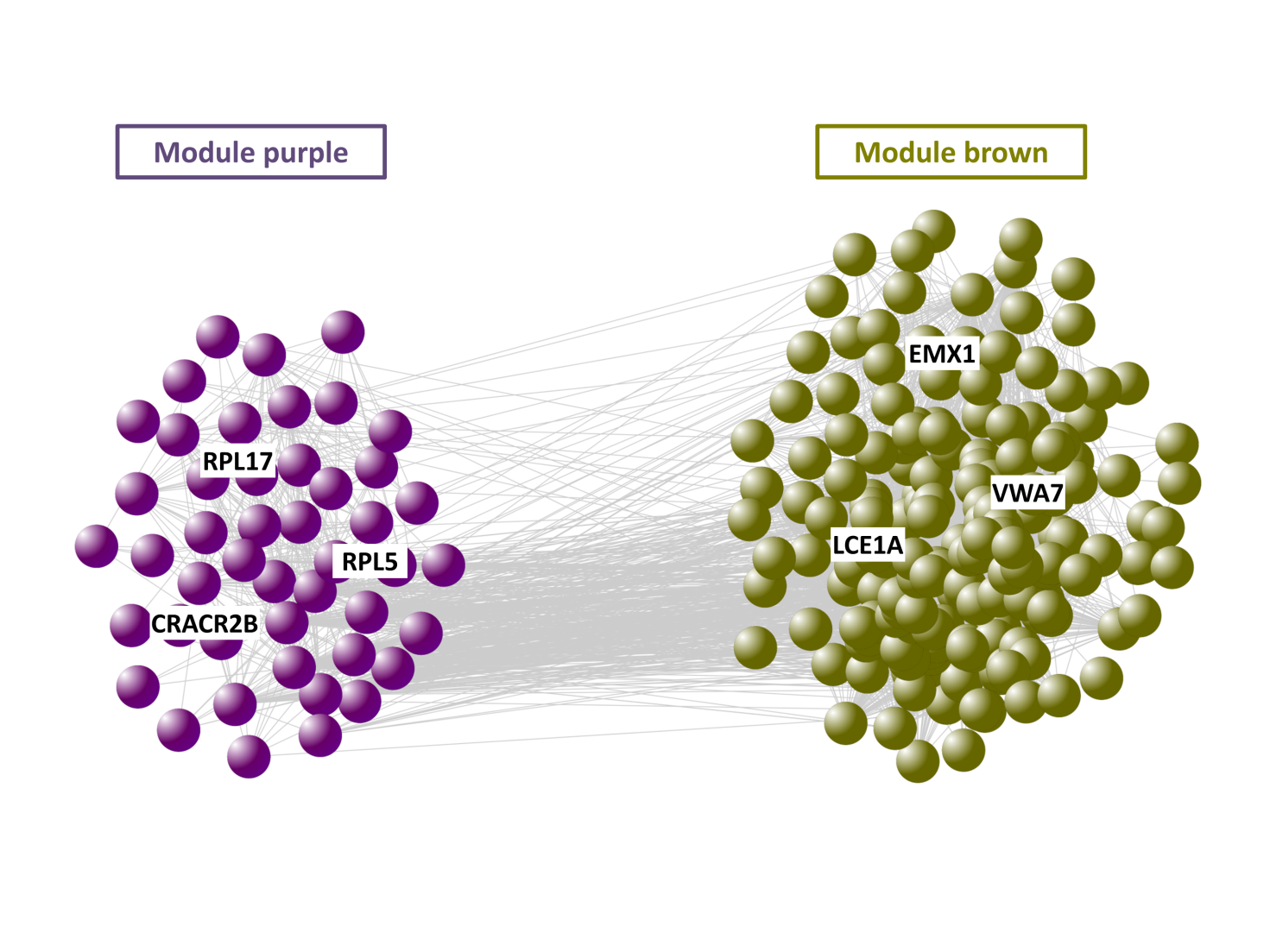


**Supplementary Figure 7. WGCNA subnetwork for modules that were significantly associated with COPD case-control status (i.e., purple module, brown module).** For each module, the top-3 genes showing the highest module membership and intramodular degree are highlighted.


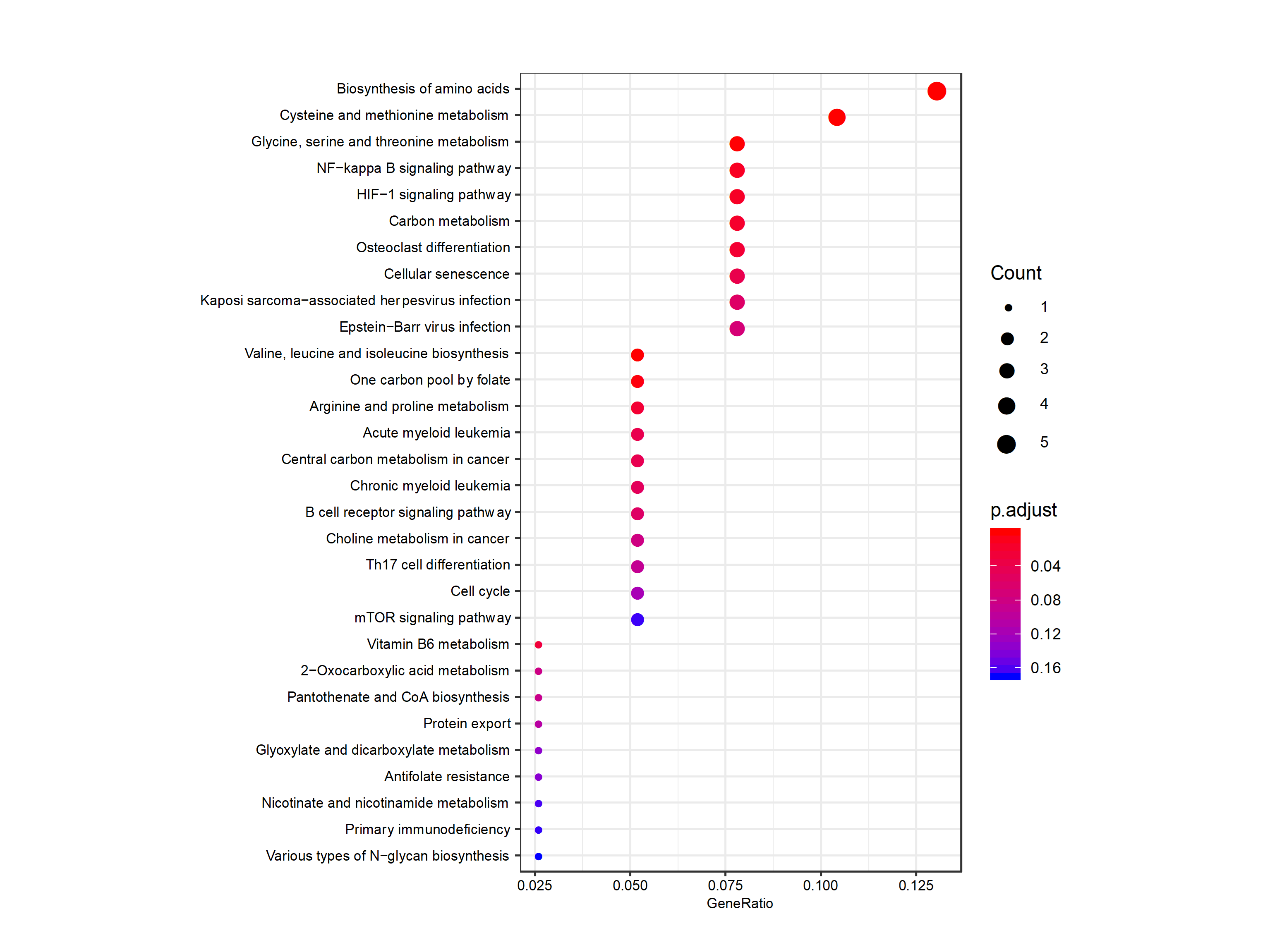


**Supplementary Figure 8. Dot plot of functional enrichment analysis for COPD switch genes.** The Y-axis reports the annotation categories (KEGG pathways) and the X-axis reports the gene ratio (i.e., the number of genes found enriched in each category over the number of total genes associated to that category). The color of the dots represents the adjusted *p*-values (FDR), whereas the size of the dots represents the number of genes found enriched in each category.


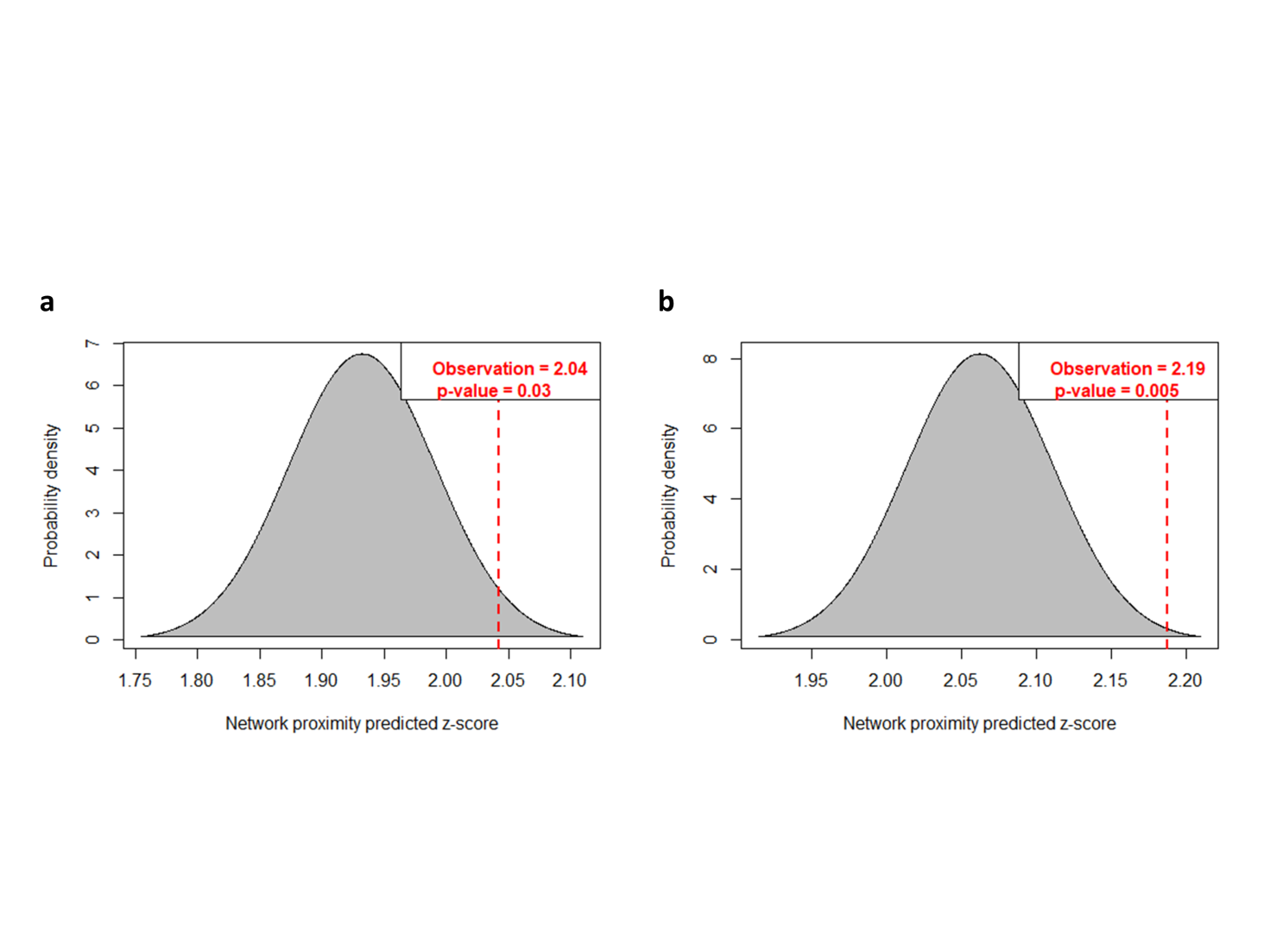


**Supplementary Figure 9. Probability distribution of the network proximity.** The network proximity was computed for the brown network module (a) and the purple network module (b) resulting from the WGCNA analysis of the COPD training set with respect to the cyan module resulting from the WGCNA analysis of the COPD test set. The dashed red lines correspond to the observed network proximity measurements. The area on the right of the dashed red lines represents the probability of observing the test statistic as high as that observed, corresponding to a p-value = 0.03 for brown module and p-value = 0.005 for purple module**.**


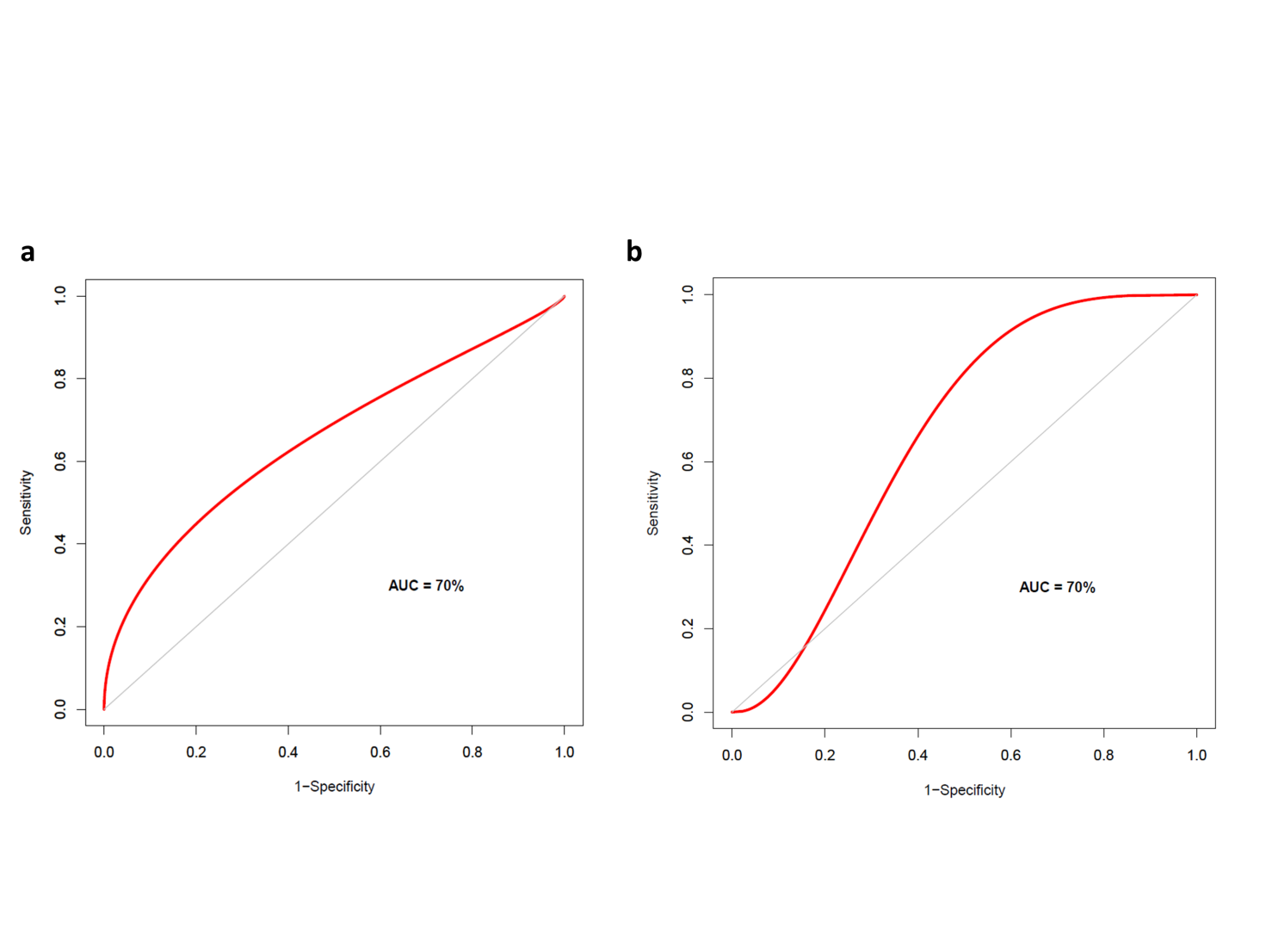


**Supplementary Figure 10. Receiver operating characteristic (ROC) curves for COPD.** The ROC curve is created for COPD training set (a) and test set (b) by plotting the true positive rate (i.e., sensitivity) placed on Y-axis against the false positive rate (i.e., 1- specificity) placed on X-axis at various threshold settings. For each threshold, we computed the true positive rate (i.e., sensitivity) placed on Y-axis, and the false positive rate (i.e., 1 - specificity) placed on X-axis. True positives are switch genes with a number of COPD GWAS interactors in the correlated network higher than a given threshold and that are annotated in COPD-related pathways (i.e., pathways that were enriched in the two lists of switch genes from training set and test set). False positives are switch genes with a number of COPD GWAS interactors in the correlated network lower than a given threshold and that are annotated in COPD-related pathways. Diagonal grey line represents the line of no-discrimination between positive class (COPD-specific genes) and negative class (genes that are not COPD-specific).

## Supplementary Tables

**Supplementary Table 1. COPD differentially expressed genes.** The table is composed of three separate sheets reporting: the statistically significant differentially expressed genes obtained for the COPD training set (GSE47460 dataset) along with their statistics (sheet 1); the differentially expressed genes of the COPD training set (GSE47460 dataset) that were previously identified as genome-wide significant COPD GWAS genes (sheet 2); and the statistically significant differentially expressed genes obtained for the COPD test set (GSE76925 dataset) along with their statistics (sheet 3).

**Supplementary Table 2.** **Node characterization in the COPD co-expression network.** The table is composed of four separate sheets reporting: all network nodes obtained for the COPD training set (GSE47460 dataset) along with their attributes and their statistics (sheet 1); bar plots reporting the number of nodes of COPD network for training set in each hub category and in each cluster (sheet 2); table reporting the immune cell-related genes for six different immune cell types (i.e., B cells, Macrophages, Monocytes, Neutrophils, Plasma cells, T cells) found in the network modules of COPD training set (sheet 3); all network nodes obtained for the COPD test set (GSE76925 dataset) along with their attributes and their statistics (sheet 4).

**Supplementary Table 3. Module membership.** The table is composed of three separate sheets reporting the list of genes belonging to each COPD network module for the training set (GSE47460 dataset) along with their module membership and their statistics.

**Supplementary Table 4. COPD switch genes.** This table is composed of six separate sheets reporting: the list of switch genes identified by SWIM for the COPD training set (GSE47460 dataset) along with their attributes and their statistics (sheet 1); the list of switch genes identified by SWIM for the COPD test set (GSE76925 dataset) along with their attributes and their statistics (sheet 2); KEGG pathways and GO Biological Process found to be associated with switch genes of the COPD training set (sheet 3-4); KEGG pathways and GO Biological Process shared between the switch genes of the COPD training set and the switch genes of the COPD test set (sheet 5-6).

**Supplementary Table 5. Nearest neighbors of COPD switch genes.** This table is composed of four separate sheets reporting for the COPD training set (GSE47460 dataset): the list of switch genes with their positively correlated GWAS genes along the columns for the COPD training set (sheet 1); the list of switch genes with their negatively correlated GWAS genes along the columns (sheet 2); the list of switch genes which negatively correlate with the GWAS genes AGER (sheet 3) and EMP2 (sheet 4).

**Supplementary Table 6. GO functional enrichment of WGCNA modules.** This table lists the top-10 GO categories enriched for each module extracted by applying WGCNA on the COPD training set.
